# Dance with robot: how employee functional experience diversity influences work wellbeing on human–robot collaboration

**DOI:** 10.3389/fpsyg.2026.1784765

**Published:** 2026-04-10

**Authors:** Yang Yang, Hongyuan Lu, Ying Li, Shuang Lin, Lingli Hu

**Affiliations:** 1 School of Mathematics, Southwestern University of Finance and Economics, Chengdu, China; 2 Institute of Western China Economic Research, Southwestern University of Finance and Economics, Chengdu, China; 3 School of International Business, Southwestern University of Finance and Economics, Chengdu, China; 4 School of Tourism Management and Exhibition, Henan University of Economics and Law, Zhengzhou, China; 5 School of Business Administration, Faculty of Business Administration, Southwestern University of Finance and Economics, Chengdu, China

**Keywords:** Conservation of Resources Theory, frontline employees, human–robot collaboration, individual adaptability, service robot, task type, work wellbeing

## Abstract

**Introduction:**

The integration of service robots into the workplace is reshaping human collaboration, while also posing a threat to employee wellbeing by introducing job uncertainty and substitution anxiety. This study examines how employees' functional experience diversity influences their work wellbeing in the context of human–robot collaboration.

**Methods:**

Grounded in Conservation of Resources Theory and Individual Adaptability Theory, we propose a moderated mediation model in which individual adaptability serves as the mediator and task type as the moderator. Two complementary studies in the hotel industry test this model. Study 1 is a survey of 175 frontline employees; Study 2 is a field experiment with 227 employees.

**Results:**

Study 1 reveals that functional experience diversity enhances work wellbeing through individual adaptability, with this indirect effect being stronger for non-routine tasks. Study 2 replicates these findings, confirming adaptability's mediating role and the amplifying effect of non-routine work.

**Discussion:**

The findings indicate that diverse functional experience constitutes a vital personal resource that fosters adaptability, which in turn promotes wellbeing, particularly in complex and uncertain work contexts. This study extends the construct of functional experience diversity from top management teams to frontline service employees, revealing the formation mechanism of employee wellbeing in the era of technological transformation. It suggests that organizations should value cross-functional experience in recruitment, cultivate employee adaptability through job rotation, and strategically match employee capabilities with task characteristics to safeguard employees' wellbeing in the wave of service automation.

## 1 Introduction

Enterprises in the Service industry are increasingly adopting service robots (Wien and Peluso, 2021). In the workplace, the collaborators of employees are shifting from humans to robots (Chang and Kim, 2022; Song and Kim, 2022). On the one hand, robot technologies free employees from tedious work by replacing repetitive tasks, thereby greatly enhancing work efficiency and job experience (Liu et al., 2025). On the other hand, technology upgrading is accompanied by potential substitutional threats (Koo et al., 2021; Vatan and Dogan, 2020). The application of robots has resulted in a reduction in job opportunities, which induces anxiety among employees and undermines their work wellbeing. Therefore, service enterprises need to pay close attention to employees' work wellbeing in human–robot teams.

In human–robot collaboration, the reasons why employees have such divergent work experiences may lie in differences in their past work experiences and personal resources. For instance, compared with employees who have long been rooted in a single position, those who have worked across multiple functional departments are likely to have accumulated richer functional experience (Cannella et al., 2008). This diverse experience is regarded as a valuable personal resource. It not only broadens employees' skill breadth and cognitive flexibility (Custódio et al., 2019; Crossland et al., 2014) but also endows them with stronger abilities to transfer knowledge and solve complex problems (Chen et al., 2021). Therefore, when facing work changes brought about by service robots, employees with rich functional experience, relying on their diverse skill reserves and broader perspectives, are more likely to perceive robots as collaborative assistants rather than substitution threats. This enables them to adapt to new responsibilities and maintain a high level of work wellbeing more easily. In contrast, employees engaged in a single position for a long time have relatively specialized knowledge structures and skill sets (Cannella et al., 2008). When robots start to replace their familiar work, this relatively singular knowledge structure and work mode may instead become a constraint, making them more prone to substitution anxiety and adaptation barriers in the face of technology shock. Consequently, this and reduces their sense of happiness (Nazareno and Schiff, 2021).

This study investigates the impact of employees' functional experience diversity on their work wellbeing in the context of human–robot collaboration, reveals the mediating role of individual adaptability, and examines the moderating effect of task types. By developing this theoretical framework, this study aims to offer a novel perspective for understanding the heterogeneity of employees' work wellbeing in human–robot collaborative settings. The findings can assist service-sector enterprises in optimizing their service robot deployment strategies, thereby mitigating the negative impacts of service robot adoption on employees and ultimately enhancing employees' wellbeing.

## 2 Literature review

### 2.1 Human–robot collaboration and its application in the hospitality industry

Human–robot collaboration is a crucial work mode in the era of artificial intelligence, with enterprises increasingly adopting robots in frontline services (Wien and Peluso, 2021). Currently, service robots are widely applied in fields such as agriculture (Vougioukas, 2019), manufacturing (Choi et al., 2025), and logistics (Belanche et al., 2020). For labor-intensive service industries, the emergence of robots has greatly improved work efficiency; however, the substitution of traditional human labor by technology poses threats to employees (Złotowski et al., 2017). Consequently, researchers have focused on the potential issues employees may face in human–robot collaboration. Studies have found that human–robot collaboration deprives employees' autonomy in the process of service, leading to feelings of frustration (Terry and Jimmieson, 2003). In addition, human–robot collaboration poses challenges to employees' work competencies, evoking a sense of substitution crisis (Złotowski et al., 2017). Moreover, when working in highly competitive organizational environments, the use of robots exacerbates this sense of crisis (Kim and Mutlu, 2014). Additionally, task characteristics influence employees' perceptions of service robots, thereby inducing resistance to working with robotic assistants (Hentout et al., 2019).

Currently, service robots have not yet acquired the communication and interaction skills required for complex emotional and social engagement (Holthöwer and Van Doorn, 2023) and thus cannot fully replace human service employees. Instead, they are only capable of performing frontline interactive service tasks that involve simple communication with customers (Wien and Peluso, 2021). For example, in the tourism industry, these tasks include food preparation, customer reception, and guidance; in the hotel industry, service robots are often used for routine duties like handling check-in procedures and assisting with luggage. Some researchers have proposed that humans and robots should work as teammates (Hvilshøj et al., 2012). By leveraging the unique strengths of both parties, human–robot teamwork can complement each other's expertise while optimizing overall team performance. In contrast to human–robot interaction, human–robot collaboration requires both parties to work together toward shared objectives within a team context (Ajoudani et al., 2018). This framework thus emphasizes the role of robots as teammates rather than tools or substitutes for humans (Hentout et al., 2019).

As service robots increasingly share the same workplace with human employees, addressing employees' attitudes toward human–robot collaboration has become particularly critical (Złotowski et al., 2017). Most service enterprises face challenges of fostering employee acceptance of robots and facilitating effective collaboration with them (Charalambous et al., 2015). While some research has begun to examine the phenomenon of employee–robot collaboration and investigate employees' attitudes toward robots as service assistants (Ivanov et al., 2019), these efforts exhibit two notable limitations. On the one hand, it overlooks individual differences among employees, for instance, how variations in prior work experience influence adaptation to emerging work modes. On the other hand, beyond work performance, greater attention should be paid to employees' psychological states in human–robot collaboration, with employee wellbeing being especially crucial. Therefore, this study explores how employees' functional experience diversity influences their work wellbeing in human–robot teams.

### 2.2 Concept and related research on functional experience diversity

Functional experience diversity is a key construct for gauging individuals' capabilities and cognition breadth (Cannella et al., 2008). It has garnered considerable academic attention in recent years as it reflects the extent of variety in the functional roles an individual has undertaken throughout their career development. In their seminal work, Cannella et al. (2008) conceptualized this construct as comprising two distinct dimensions. First is dominant functional diversity at the team level, referring to the degree of heterogeneity in top management teams composed of specialists with diverse professional backgrounds. Second is internal functional diversity at the individual level, which measures the breadth of cross-functional work experience possessed by individual members. This latter perspective illuminates disparities in the capability structures of individual employees, directly distinguishing between those with rich multi-functional experience and those who specialize in a single functional domain. In essence, functional experience diversity not only reflects the breadth of an individual's knowledge and skills but also embodies the flexibility of their cognitive frameworks and their comprehensive abilities to address complex challenges (Custódio et al., 2019).

Research on the outcomes of functional experience diversity primarily falls into two levels: individual and team. On the individual level, studies have shown that functional experience diversity endows individuals with significant advantages in complex and uncertain environments (Chen et al., 2021; Crossland et al., 2014). Such diverse functional experience broadens their breadth of perspective (Chen et al., 2021), expands their diverse range of skills and cognitive flexibility (Crossland et al., 2014), and enables them to analyze problems from multiple angles. Furthermore, managers with multi-functional work experience possess richer social resources and stronger capabilities to integrate resources (Monge, 1987). Consequently, they are more likely to respond to environmental changes with agility and make informed decisions (Bunderson and Sutcliffe, 2002). At the team level, the internal functional diversity of members facilitates information sharing and integration within the team (Bunderson and Sutcliffe, 2002), mitigates biases and parochial mindsets, and thereby enhances organizational performance. In contrast, while specialists possess in-depth knowledge and skills in specific domains, their relatively singular knowledge structures and work patterns (Cannella et al., 2008) may hinder their ability to adapt to changes of the organizational environment quickly.

Despite significant advancements in research on functional experience, notable limitations remain. Existing literature has predominantly focused on the top management level (Cannella et al., 2008), exploring its impact on organizational innovation or strategic decision-making; however, discussions on how functional experience diversity influences employees' internal psychological states remain underdeveloped. Accordingly, this study shifts the research focus to frontline service employees in the context of human–robot collaboration, aiming to investigate the intrinsic relationship between employees' functional experience diversity and wellbeing.

## 3 Research design and hypothesis development

### 3.1 The impact of functional experience diversity on work wellbeing in human–robot collaboration

Work wellbeing refers to employees' overall state of happiness in the workplace (Warr, 2006). It integrates both work-related subjective wellbeing and psychological wellbeing, representing employees' comprehensive evaluation and emotional experience of all aspects of their jobs (Van Horn et al., 2004). Drawing on the Conservation of Resources (COR) Theory, individuals tend to acquire, retain, and protect the resources they value, and work wellbeing is largely determined by the extent to which individuals gain or lose resources in the workplace (Hobfoll et al., 2018). Restructuring of work processes, changes in skill requirements, and potential substitution threats (Koo et al., 2021; Vatan and Dogan, 2020) by the introduction of service robots, may induce employees to perceive resource depletion or threats, thereby undermining their work wellbeing.

However, employees do not passively succumb to such impacts; their prior career experiences provide them with varying levels of personal resources. As a reflection of individuals' capabilities and cognitive breadth (Cannella et al., 2008), functional experience diversity serves as a critical resource for coping with environmental uncertainty. Specifically, employees with high functional experience diversity have accumulated a broader range of knowledge, skills, and cross-domain experience throughout their careers (Custódio et al., 2019; Crossland et al., 2014). Such diverse resource reserves equip them with greater psychological resilience and adaptive flexibility when confronting changes brought about by human–robot collaboration. They are less likely to experience substitute anxiety due to single skills; instead, they may perceive new technologies as opportunities to expand their diverse capabilities, thereby benefiting from the dynamic balance of resources and maintaining high levels of work wellbeing. In contrast, employees with relatively low functional experience diversity have personal resources deeply tied to specific positions (Cannella et al., 2008). When robots begin to take over their core tasks, these employees are more prone to feeling resource loss and developing career insecurity (Schweiger and Denisi, 1991). This resource depletion and stress perception triggered by technological impacts will ultimately significantly reduce their work wellbeing. Accordingly, we propose the following hypothesis:

H1: In the context of human–robot collaboration, employees with high functional experience diversity exhibit higher work wellbeing than those with low functional experience diversity.

### 3.2 The impact of individual adaptability on work wellbeing

Individual adaptability refers to an individual's tendency to adjust their cognition, behaviors, and emotions to adapt to changes when facing new situations (Ployhart and Bliese, 2006; Hua et al., 2019). As a key personal trait influencing employees' work experiences (Ma et al., 2025), individual adaptability has been conceptualized by Ployhart and Bliese (2006) based on the Individual Adaptability Theory into five dimensions: work-stress adaptability, uncertainty adaptability, learning adaptability, problem-solving adaptability, and inter-personal adaptability. In addition, individual adaptability does not possess only a single trait in itself but a composite variable consisting of a series of deeper-level knowledge, skills, abilities, and other characteristics (KSAOs). Among these KSAOs, an individual's prior experience is regarded as one of the core sources of adaptability (Ployhart and Bliese, 2006).

This study posits that employees' work experience is essentially a process of shaping key cognitive KSAOs. These experiences compel employees to continuously confront and address knowledge from diverse domains, different work processes, and varied ways of thinking, thereby fostering their cognitive capabilities (Custódio et al., 2019). Thus, in line with the Individual Adaptability Theory, employees with richer functional experience diversity possess a more robust cognitive capabilities, that is foundation in KSAOs, and consequently exhibit greater individual adaptability.

In the context of human–robot collaboration, the introduction of service robots not only changes the work environment but also introduces uncertainty that threatens employees' sense of control and competence, thereby depleting their psychological resources (Henkel et al., 2020). Employees with high adaptability can proactively leverage their cognitive and behavioral resources to manage such threats and adapt to changes in the work environment. This proactive adaptation to environmental changes enables them to regain a sense of control over their work and self-efficacy (Bandura, 1999), effectively preventing the depletion of psychological resources and thereby maintaining high levels of work wellbeing. In contrast, employees with insufficient adaptability may experience stress due to the uncertainty brought by technology, leading to reduced efficiency and even job burnout (Schweiger and Denisi, 1991). Accordingly, this study proposes the following hypothesis:

H2: In the context of human–robot collaboration, individual adaptability mediates the relationship between functional experience diversity and work wellbeing. Specifically, compared with employees with low functional experience diversity, high functional experience diversity helps employees develop stronger individual adaptability, which in turn enhances their work wellbeing.

### 3.3 The moderating role of task type

Task types can generally be categorized into routine tasks and non-routine tasks (Gladstein, 1984). Routine tasks are highly repetitive and can be performed according to explicit rules, while non-routine tasks involve complex non-programmed decision-making, interpersonal communication, and innovative problem-solving (Autor et al., 2003). Drawing on the job demands–resources (JD-R) model, different work tasks can be regarded as job demands of distinct natures, thereby influencing the processes of employees' resource activation and depletion (Bakker et al., 2023; Cavanaugh et al., 2000).

In the context of routine tasks, work is repetitive, simple, and highly procedural. Against the backdrop of human–robot collaboration, robots are designed to perfectly substitute for such tasks (Autor et al., 2003). This renders the tasks themselves unable to provide scenarios for exerting individual adaptability and may be perceived by employees as hindrance demands. Hindrance demands are defined as negative factors that impede the achievement of personal goals and merely increase resource depletion (Cavanaugh et al., 2000). Employees may view robots as a source of monotonous work, skill atrophy, and even job threats. In this context, employees' psychological resources are primarily allocated to coping with anxiety and career insecurity rather than engaging in proactive adaptation. Thus, even if employees possess high individual adaptability, these resources are either deprived of room for exertion or inhibited by hindrance demands, significantly attenuating the positive impact on work wellbeing.

In contrast, in the context of non-routine tasks, work is characterized by uncertainty, complexity, and challenge. According to the job demands–resources (JD-R) model, such tasks constitute challenge demands. While challenge demands require employees to expend effort, they are also perceived as opportunities that foster personal growth, learning, and future gains (Cavanaugh et al., 2000). In the context of human–robot collaboration, non-routine tasks mean that robots may handle information processing, while employees are required to make complex judgments and decisions. At this point, employees' individual adaptability can be fully exerted. Employees with high adaptability can be motivated by such challenges: they proactively leverage their adaptive resources to learn, explore, and optimize human–robot collaboration processes, as well as solve unexpected problems. This successful activation and deployment of resources generate a strong sense of achievement and work competence, thereby enabling individual adaptability to be most effectively translated into higher work wellbeing. According, we propose the following hypothesis:

H3: In the context of human–robot collaboration, task type moderates the impact of individual adaptability on work wellbeing. Specifically, compared with routine tasks, the positive effect of individual adaptability on work wellbeing is stronger in non-routine tasks.

**Figure 1:**
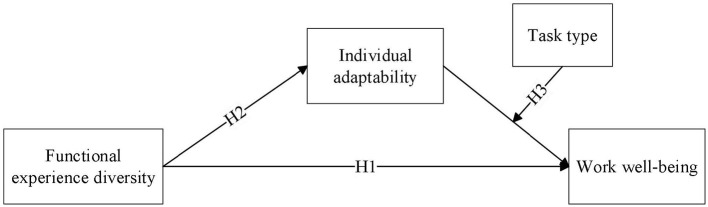
Theoretical model.

Based on the three research hypotheses, this study proposes the theoretical model illustrated in Figure 1. To test these proposed hypotheses, this study designs two empirical studies. Hotels are typical service-sector enterprises, and service robots have been widely adopted in hotel operations. Given these characteristics, this study selects hotels as the research context for data collection. Study 1 employs a questionnaire survey among frontline hotel employees to examine the main impact of employees' functional experience diversity on work wellbeing, the mediating effect of individual adaptability, and the moderating effect of task type. Study 2 adopts a field experiment to replicate research hypotheses.

## 4 Study 1

### 4.1 Data collection and sample description

Study 1 employs a questionnaire survey method, aiming to examine the main effect of employees' functional experience diversity on work wellbeing, the mediating effect of individual adaptability, and the moderating effect of task type. In collaboration with the hotel industry association, we recruited six hotels that had adopted service robots, surveying a total of 216 frontline employees. All participating employees were in positions requiring collaboration with service robots to complete tasks, such as front desk staff who collaborate with robots to handle check-in procedures, housekeeping staff who work with robots to fulfill item delivery, and concierge staff who coordinate with robots to perform luggage handling.

The study involving human participants were reviewed and approved by the Faculty of Business Administration at Southwestern University of Finance and Economics (Approval No: 2024SBA024). All procedures involving human participants in this study complied with the ethical standards of the institutional research committee.

The data for Study 1 were collected from September to October 2024. This period excluded the summer tourism peak (July–August) in the research site (Chengdu, Sichuan Province, China) as well as the post-holiday adjustment period immediately following the National Day Golden Week (early October). This timing ensured a relatively stable operational status for hotels and reduced the potential impact of seasonal employment fluctuations on sample representativeness. The hotel industry in Chengdu is well-developed, with a diversified customer base dominated by business and leisure travelers, which guarantees the typicality and diversity of the research context.

To mitigate the potential impact of common method bias on the validity of the research findings, a two-wave questionnaire survey was adopted for data collection in Study 1. Prior to data collection, all potential participants were provided with a detailed information statement that explicitly elaborated on the research objectives, the anonymity and confidentiality of data collection, the voluntary nature of participation, and the right to withdraw from the study at any time. After fully understanding the study details, all participants signed a written informed consent form. In Wave 1, employees were asked to report their demographic characteristics, self-rated functional experience diversity, individual adaptability, and the task types associated with their daily job responsibilities. The questionnaire data were collected anonymously; all personal information was coded and de-identified prior to data analysis, and the data were used solely for this study. In Stage 2, employees completed a self-assessment of their work wellbeing.

After excluding 41 questionnaires with missing values or outliers, a final sample of 175 valid responses was retained for subsequent analyses. The demographic profile of the sample was as follows:

Gender: males accounted for 44.6%, while females made up 55.4%.

Age: participants were predominantly distributed across three age groups: 30–35 years old (46.9%), 25–30 years old (29.7%), and 36–40 years old (13.1%).

Educational level: the majority of respondents held either a junior college degree (34.3%) or a bachelor's degree (54.3%).

Organizational tenure: most employees had 2–3 years of working experience in their current organizations, accounting for 41.1% of the total sample.

Departments: participants were mainly from the Food and Beverage Department (23.4%), Housekeeping Department (19.4%), and Sales and Marketing Department (13.7%).

### 4.2 Measurement instruments

Study 1 focused on four focal variables and five control variables, with all scales being well-established and previously validated in top academic journals.

Functional experience diversity was measured using a two-item scale adapted from Cannella et al. (2008). A sample item was: “I have worked across multiple distinct functional departments in my job.” Work wellbeing was assessed with a six-item scale adapted from Zheng et al. (2015) and contextualized to human–robot collaboration. A sample item was: “When collaborating with robots, I feel satisfied with my job responsibilities.” Individual Adaptability was measured using a 14-item scale adapted from Ployhart and Bliese (2006). A sample item is: “When collaborating with robots, I tend to overreact during peak business periods in the hotel.” Task type was evaluated with a five-item scale adapted from Podsakoff and MacKenzie (1994), with all items scored in reverse. A sample item was “Most of the tasks I complete in collaboration with robots are repetitive” (Table 1). All the aforementioned items were rated on a 7-point Likert scale (1 = strongly disagree, 7 = strongly agree). To eliminate potential confounding effects, we controlled five demographic variables of the employees: gender, age, education, work tenure, and department.

**Table 1:** Summary of measurement scale.

Construct	Item	Item content	Source
Functional experience diversity	FED1	I have worked across multiple distinct functional departments in my job	Cannella et al., (2008)
	FED2	In my past work, I have worked in frontline service positions across multiple different departments	
Task type	TT1	Most of the tasks I complete in collaboration with robots are repetitive	Podsakoff and MacKenzie, (1994)
	TT2	I perform the same type of work each day when collaborating with robots	
	TT3	The work I do with robots changes little from day to day	
	TT4	The tasks I complete with robots are fairly simple	
	TT5	I follow the same set of steps to complete most of my work with robots	
Individual adaptability	IA1	When collaborating with robots, I tend to overreact during peak business periods in the hotel	Ployhart and Bliese, (2006)
	IA2	When collaborating with robots, excessive work pressure at the hotel makes me feel inadequate	
	IA3	When tasks with robots are tightly scheduled, I easily become irritable	
	IA4	When collaborating with robots, heavy workload at the hotel usually makes me feel stressed	
	IA5	When under great pressure while collaborating with robots, I often cry or become angry	
	IA6	When collaborating with robots, I need tasks to be black and white, clear and unambiguous	
	IA7	When collaborating with robots, unpredictability of tasks frustrates me	
	IA8	When collaborating with robots, I can make effective decisions even without all relevant information	
	IA9	When the work situation with robots is stable, I perform best	
	IA10	When encountering unexpected situations with robots, I can adjust quickly	
	IA11	I can adapt to constantly changing collaboration situations with robots	
	IA12	I perform well when work with robots is unstable	
	IA13	I have difficulty coping with changing conditions when collaborating with robots	
	IA14	When collaborating with robots, I adjust my plans according to changing conditions	
Work wellbeing	WWB1	When collaborating with robots, I feel satisfied with my job responsibilities	Zheng et al., (2015)
	WWB2	Overall, I am satisfied with my current work state	
	WWB3	When collaborating with robots, I always find ways to enrich the job content	
	WWB4	I have found real enjoyment in working with robots	
	WWB5	Working with robots is a meaningful experience for me	
	WWB6	I am basically satisfied with my achievements in working with robots	

### 4.3 Reliability and validity tests

To verify the internal consistency of the scales, reliability analyses were conducted sequentially for the measures of functional experience diversity, work wellbeing, individual adaptability, and task type (Table 1). The Cronbach's α coefficients for all constructs exceeded the minimum threshold of 0.700, indicating that the scales possessed satisfactory reliability (DeVellis, 1991).

Confirmatory factor analysis (CFA) was performed using Amos 22.0. The model demonstrated a good fit to the data: χ^2^/d*f* = 1.423, RMSEA = 0.049 (<0.080), CFI = 0.966 (>0.900), and TLI = 0.963 (>0.900). The average variance extracted (AVE) values for all constructs were greater than 0.5, which confirmed that the measurement model had excellent convergent validity. Furthermore, the square root of the AVE for each construct was larger than its correlation coefficients with other constructs, providing additional evidence of good discriminant validity for the measurement model.

### 4.4 Hypotheses testing

#### 4.4.1 Main effect analysis

Hierarchical regression analyses were conducted using SPSS 27.0 to test the main effect (see Table 2). First, control variables—gender, age, education, work tenure, and department—were entered into the model to examine their impact on work wellbeing (Model 1). Second, functional experience diversity (FED) was added as the independent variable in Model 2. Results of Model 2 indicated that compared with Model 1, the change in *R*-squared was significant (Δ*R*^2^ = 0.162, *F* change = 34.605, *p* < 0.001). Specifically, functional experience diversity had a significant positive effect on employees' work wellbeing (β = 0.407, *t* = 5.883, *p* < 0.001). Thus, Hypothesis 1 (H1) was supported.

**Table 2:** Results of reliability and validity test.

Variable	*M*	SD	Cronbach's α	CR	AVE	1	2	3	4
FED	3.89	1.790	0.829	0.830	0.709	[0.842]			
WWB	4.68	1.465	0.901	0.918	0.651	0.420^***^	[0.807]		
IA	4.33	1.593	0.972	0.972	0.710	0.419^***^	0.597^***^	[0.842]	
TT	3.67	1.548	0.905	0.906	0.658	0.319^***^	0.480^***^	0.549^***^	[0.811]

*** p < 0.001; values in brackets were the square roots of average variance extracted (AVE); FED, functional experience diversity; WWB, work wellbeing; IA, individual adaptability; TT, task type.

#### 4.4.2 Mediation effect analysis

**Figure 2:**
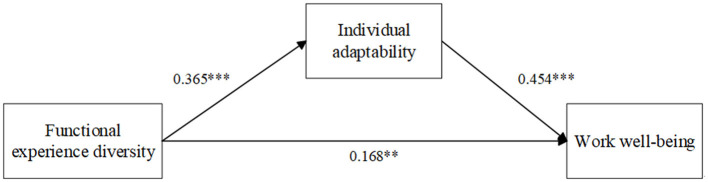
Mediation model results in study 1. **p < 0.01, ***p < 0.001.

The bootstrap method (PROCESS Macro, Model 4, *n* = 5,000) was employed to test the mediating role of individual adaptability (IA) (Hayes, 2013). Results revealed a significant indirect effect of functional experience diversity on work wellbeing (WWB) via individual adaptability [indirect effect = 0.165, 95% CI = (0.094, 0.242), not include zero; Figure 2]. Hypothesis 2 (H2) was therefore supported.

Additionally, the direct effect of functional experience diversity on work wellbeing remained significant [direct effect = 0.168, 95% CI = (0.060, 0.276), not include zero]. These findings confirmed that individual adaptability partially mediates the relationship between functional experience diversity and work wellbeing (see Tables 3, 4).

**Table 3:** Results of hierarchical regression.

Varibles	Model 1	Model 2
Gender	0.570^*^ (2.539)	0.448^*^ (2.177)
Age	0.214 (1.653)	0.159 (1.345)
Education	0.130 (0.808)	0.188 (1.283)
Department	−0.070 (−1.265)	−0.064 (−1.259)
Work tenure	−0.081 (−0.692)	−0.093 (−0.865)
Functional experience diversity		0.333^***^ (5.883)
*R* ^2^	0.052	0.214
Δ*R*^2^		0.162
*F*	1.868	7.634^***^
*F change*		34.605^***^

* p < 0.05,

*** p < 0.001; t-values are presented in parentheses.

**Table 4:** Results of mediation analysis.

Variables	IA		WWB		WWB	
	β	***t***	β	***t***	β	***t***
FED	0.365^***^	6.013	0.168^**^	3.064	0.333^***^	5.883
IA			0.454^***^	7.186		
Gender	0.454^*^	2.060	0.242	1.325	0.448	2.177
Age	0.265^*^	2.091	0.039	0.371	0.159	1.345
Education	0.389^*^	2.476	0.012	0.090	0.188	1.283
Department	−0.039	−0.708	−0.046	−1.042	−0.064	−1.260
Work tenure	−0.170	−1.478	−0.016	−0.166	−0.093	−0.865
*R^2^*	0.238		0.400		0.214	
*F*	8.739^***^		15.893^***^		7.634^***^	

* p < 0.05,

** p < 0.01,

*** p < 0.001 (two-tailed tests), FED, functional experience diversity; WWB, work wellbeing; IA, individual adaptability.

#### 4.4.3 Moderating effect analysis

The bootstrap method (PROCESS Macro, Model 14, *n* = 5,000) was employed to test the moderating effect of task type (TT) (Hayes, 2013). Results demonstrated a significant interactive effect of individual adaptability (IA) and task type on work wellbeing (WWB) (β = 0.102, LLCI = 0.036, ULCI = 0.167, excluding zero).

**Figure 3:**
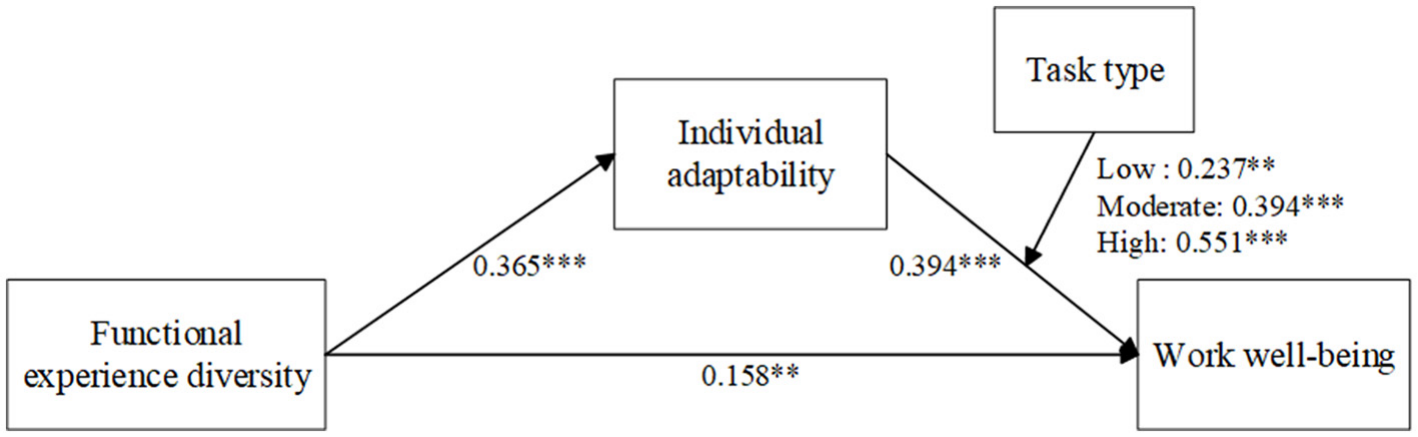
Moderated mediation model results in study 1. **p < 0.01, ***p < 0.001.

To further explore the nature of this moderation effect, simple slope analyses was conducted by categorizing task routineness (the operationalized dimension of task type) into three levels: low (*M* – 1SD), moderate (*M*), and high (*M* + 1SD). The findings are as follows: at the low level of task routineness (M – 1SD), the mediating effect of individual adaptability was significant (β = 0.086, LLCI = 0.012, ULCI = 0.171, excluding zero). At the moderate level of task routineness (*M*), the mediating effect of individual adaptability remained significant (β = 0.144, LLCI = 0.071, ULCI = 0.222, excluding zero). At the high level of task routineness (*M* + 1SD), the mediating effect of individual adaptability was not only significant but also exhibited a larger effect size (β = 0.201, LLCI = 0.114, ULCI = 0.286, excluding zero). In sum, these results indicate that the positive effect of individual adaptability on work wellbeing strengthens as the level of task routineness (i.e., task complexity) of employees' positions increases (Figure 3). Therefore, Hypothesis 3 (H3) was supported.

### 4.5 Discussion

Study 1 employed a questionnaire survey to capture the practical perceptions of frontline hotel employees engaged in human–robot collaboration and tested the proposed research hypotheses based on their actual work experiences. The results confirmed that employees' functional experience diversity exerts a positive impact on their work wellbeing. Specifically, during human–robot collaboration, employees with high functional experience diversity reported significantly higher levels of work wellbeing compared to those with low functional experience diversity. Further analyses revealed that individual adaptability mediates the relationship between functional experience diversity and work wellbeing. Moreover, the effect of functional experience diversity on work wellbeing was greater under non-routine tasks than under routine tasks, confirming the moderating role of task type.

Although Study 1 supported all the proposed hypotheses, it is confined to examining static correlational relationships within the human–robot collaboration context. To address this limitation, Study 2 will adopt a field experiment approach to examine the influence mechanism linking employees' functional experience diversity on work wellbeing in a more realistic and dynamic setting. Additionally, it will further examine whether task type moderates the effect of collaboration competence on work wellbeing.

## 5 Study 2

### 5.1 Research design and participants

Study 2 adopted a 2 (functional experience diversity: low vs. high) × 2 (task type: routine vs. non-routine) between-subjects design. The experiment was conducted at three branches of a hotel chain that has integrated robotic systems across all its departments to support service operations. All three branches are in Chengdu, Sichuan Province, China, and belong to the same chain. Selecting hotel branches from the same brand with similar geographical locations helps control for potential confounding variables, such as organizational culture, management processes, and customer mix, thereby enhancing the internal validity of the study. Participants were frontline employees of this hotel brand, with a final valid sample of 227 respondents (47.1% female).

The study involving human participants was reviewed and approved by the Faculty of Business Administration at Southwestern University of Finance and Economics (Approval No.: 2024SBA024). All procedures involving human participants were conducted in accordance with the ethical requirements of the institutional research ethics committee.

### 5.2 Procedure and measures

The field experiment for Study 2 was conducted from April to May 2025 and lasted 4 weeks. This period coincided with the spring shoulder season for the hotel industry, during which hotel occupancy rates were moderate. This timing ensured that employees had sufficient opportunities to collaborate with robots on tasks, while avoiding the excessive workloads of peak seasons that could potentially interfere with experimental control.

Participants were randomly assigned to different hotel departments (e.g., front desk, housekeeping, F&B department). Prior to the study, all participants were informed of the nature, duration, and procedures, including the requirement to collaborate with service robots for 1 week and subsequently complete a research questionnaire. Participants were explicitly informed that their participation was completely voluntary and that their decision to participate or withdraw at any time would not affect their job evaluations or career development within the hotel. After fully understanding all the information, each participant signed a written informed consent form. Subsequently, they were then required to collaborate with robots to perform their tasks over a 1-week period. One week later, participants were invited to complete a questionnaire consisting of scales measuring task type (α = 0.893), individual adaptability (α = 0.914), and work wellbeing (α = 0.905), along with demographic information (consistent with those used in Study 1). All collected data were anonymized, used exclusively for the purposes of this study, and not disclosed to any third party.

### 5.3 Manipulation check and results

#### 5.3.1 Manipulation check

A one-way analysis of variance (ANOVA) was conducted to verify the effectiveness of task type manipulation. Results indicated that the task type score was significantly higher in the non-routine task group than in the routine task group [*M*_non − routine_ = 5.52, SD = 0.518 vs. *M*_routine_ = 4.90, SD = 0.775; *F*_(1,225)_ = 50.595, *p* < 0.001, η^2^ = 0.184]. This result showed that the manipulation of the moderator (task type) was successful.

#### 5.3.2 Main effects

Another one-way ANOVA was performed to test the main effect of functional experience diversity on work wellbeing. As expected, employees with high functional experience diversity reported significantly higher work wellbeing than those with low functional experience diversity [*M*_high_ = 5.43, SD = 0.842 vs. *M*_low_ = 4.22, SD = 0.786; *F*_(1,225)_ = 124.212, *p* < 0.001, η^2^ = 0.356]. This result revalidated Hypothesis 1 (H1).

Additionally, a one-way ANOVA revealed that employees with high functional experience diversity exhibited significantly higher individual adaptability compared to those with low functional experience diversity [*M*_high_ = 5.53, SD = 0.999 vs. *M*_low_= 4.349, SD = 0.953; *F*_(1,225)_ = 83.440, *p* < 0.001, η^2^ = 0.271]. This result further supported Hypothesis 2 (H2).

#### 5.3.3 Moderation effect analysis

**Figure 4:**
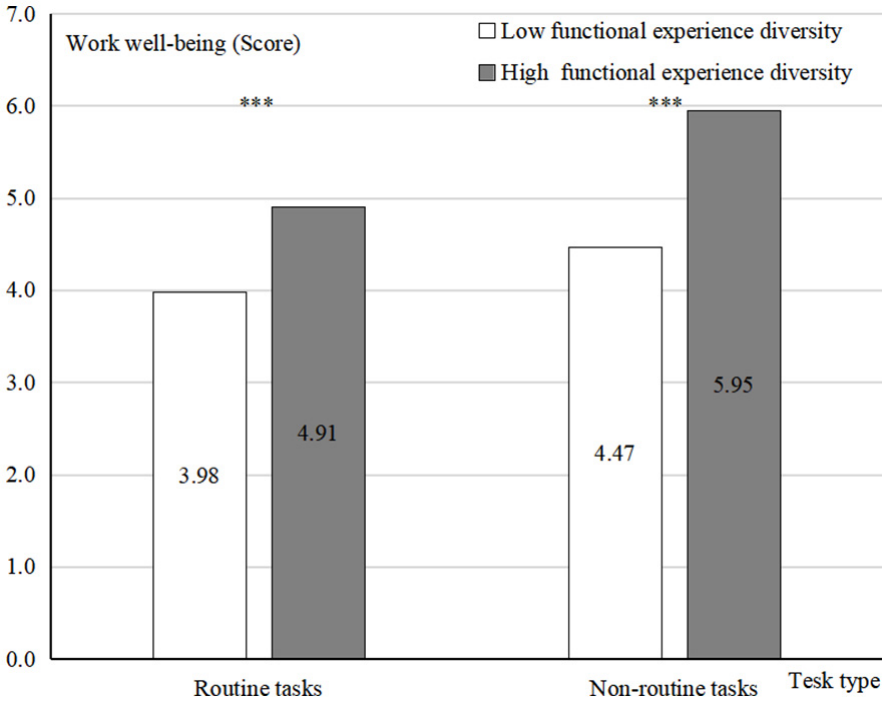
Result of moderating effect. ***p < 0.001.

To test Hypothesis 3 (H3), a two-way ANOVA (coded as 0 = low reference group, 1 = high experimental group) revealed a significant interaction effect between functional experience diversity and task type on work wellbeing [*F*_(1,223)_ = 8.770, *p* = 0.003, η^2^ = 0.038; see Figure 4].

Further intergroup analysis was conducted.

For routine tasks, employees with high functional experience diversity reported significantly higher work wellbeing than those with low functional experience diversity [*M*_high_ = 4.91, SD = 0.806 vs. *M*_low_ = 3.98, SD = 0.643; *F*_(1,112)_ = 46.330, *p* < 0.001, η^2^ = 0.293].

For non-routine tasks, employees with high functional experience diversity significantly outperformed on work wellbeing than those with low functional experience diversity [*M*_high_ = 5.95, SD = 0.469 vs. M_low_ = 4.47, SD = 0.843; *F*_(1,111)_ = 136.326, *p* < 0.001, η^2^ = 0.551].

**Figure 5:**
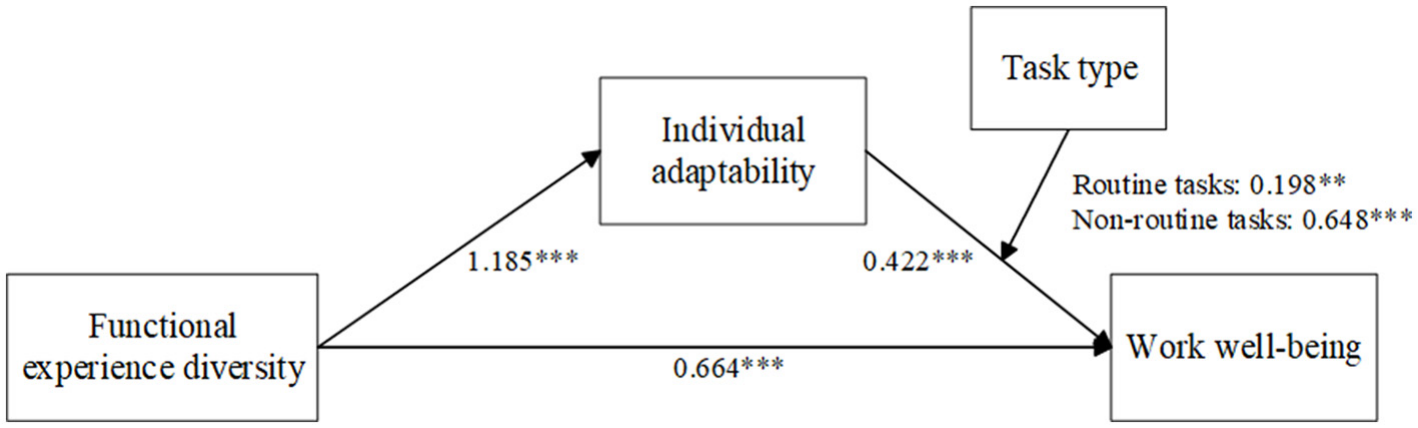
Moderated mediation model results in study 2. **p < 0.01, ***p < 0.001.

To further confirm the mediation effect of task type, a bootstrap analysis (Model 14, in PROCESS) was conducted. Results indicated a significant interactive effect of individual adaptability and task type on work wellbeing (β = 0.450, LLCI = 0.277, ULCI = 0.623, not include zero). When performing routine tasks: the mediating effect of individual adaptability was significant (β = 0.234, LLCI = 0.028, ULCI = 460, not include zero). When performing non-routine tasks: the mediating effect of individual adaptability remained significant and had a larger effect size (β = 0.768, LLCI = 0.480, ULCI = 1.068, not include zero; see Figure 5). Collectively, these findings revalidated Hypothesis 3 (H3).

### 5.4 Discussion

Study 2 adopted a field experiment to replicate the entire model, which enhanced the robustness of the research findings. The test results were consistent with the hypotheses proposed in this study: employees' functional experience diversity positively influences their work wellbeing, and individual adaptability mediates this relationship. This indicates that as a form of resource heterogeneity among employees, functional experience diversity ultimately improves their work wellbeing by enhancing their adaptability. The moderating effect of task type shows that compared with routine tasks, the effect of individual adaptability on wellbeing is more significant when handling non-routine tasks. In other words, in human–robot collaboration, employees with higher functional experience diversity are more likely to gain wellbeing in positions when performing non-routine tasks.

## 6 General discussion

### 6.1 Conclusions

Service robots are driving the transformation of the service industry, rendering human–robot collaboration a prevalent and normalized work mode. However, employees demonstrate significant heterogeneity in their responses to this collaborative model, which in turn affects their work experience. Given the critical impact of work wellbeing on employee performance, job satisfaction, and retention intention (Eliyana and Ma'arif, 2019), identifying the factors that help employees maintain a high level of work wellbeing in the context of human–robot collaboration has become an important issue for service organizations. This research, through two complementary studies—a questionnaire survey and a field experiment—investigated the facilitating role of functional experience diversity in promoting employees‘ work wellbeing within human–robot collaboration settings.

First, in line with the tenets of conservation of resources (COR) theory (Hobfoll et al., 2018), this study confirms that employees' functional experience diversity acts as a critical personal resource for sustaining work wellbeing in the human–robot collaboration context. While prior research has established that technology-induced changes can trigger resource depletion and substitution anxiety (Koo et al., 2021; Vatan and Dogan, 2020), our findings reveal that compared with employees who have long specialized in a single position, those with cross-departmental rotation experience exhibit significantly higher work wellbeing. This finding validates our core hypothesis that functional experience diversity itself acts as a robust psychological buffer: when confronting work-flow restructuring and potential substitution threats brought by service robots, employees with diverse functional experiences possess greater resource reserves to sustain their wellbeing.

Second, drawing upon individual adaptability theory (Ployhart and Bliese, 2006), our results demonstrate that individual adaptability plays a mediating role in the relationship between employees' past functional experience diversity and their current work wellbeing. Employees' past functional experiences do not directly translate into wellbeing; instead, they act as an antecedent that exerts influence by shaping employees' intrinsic capacity to cope with changes. This finding aligns with the theoretical proposition that adaptability is rooted in deeper-level knowledge, skills, abilities, and other characteristics (KSAOs) accumulated through experience (Ployhart and Bliese, 2006). Specifically, cross-departmental rotation experiences prompt individuals to continuously restructure their cognitive and behavioral patterns in different work scenarios, and such repeated practice hones their adaptability. Thus, the reason why employees with high functional experience diversity can maintain higher work wellbeing in human–robot collaboration is largely that this internalized strong adaptability enables them to restructure the uncertainty brought by technology into opportunities for career development, thereby effectively mitigating the psychological impact caused by environmental changes.

Third, this study identifies critical boundary conditions underlying the relationship between individual adaptability and work wellbeing, which aligns with the job demands–resources (JD-R) Model (Bakker et al., 2023; Cavanaugh et al., 2000). Our results demonstrate that task type plays a moderating role in the relationship between functional experience diversity and work wellbeing. The individual adaptability accumulated through employees' past functional experiences does not exert a consistent effect across all contexts. Results from both studies consistently demonstrate that the positive impact of individual adaptability on work wellbeing is significantly amplified in non-routine tasks. In such contexts, where tasks are characterized by complexity and uncertainty (Autor et al., 2003), employees with high adaptability can fully exert their capabilities in problem-solving and coping with uncertainty, thereby gaining a sense of competence and achievement. By contrast, the mediating effect of individual adaptability is attenuated in routine tasks. This phenomenon implies that when an employee with high adaptability is confined to a routine position in human–robot collaboration, their accumulated adaptive advantages will have no room to be exerted; instead, the mismatch between the employee's adaptability and the human–robot collaborative tasks inhibits their work wellbeing.

### 6.2 Theoretical contributions

First, this study makes an innovative theoretical bridge between research domains of human resource management (HRM) and human–robot collaboration. Prior research on functional experience diversity has primarily focused on the CEO and top management team (TMT) levels, with attention to its impacts on macro-level outcome variables such as organizational innovation (Pan et al., 2025) and firm performance (Monge, 1987). In contrast, this study extends this theoretical construct to frontline employees in the service industry. Empirical results demonstrate that functional experience diversity (FED) serves not merely as a strategic resource for senior executives but also as a critical personal resource for frontline employees to sustain their micro-level psychological wellbeing (e.g., individual adaptability and work wellbeing) when facing technological changes (e.g., human–robot collaboration). Thus, this finding significantly expands the application contexts of the functional experience diversity (FED) construct.

Second, this study unpacks the “black box” between functional experience diversity and employees' work wellbeing. Drawing on conservation of resources (COR) theory and individual adaptability theory, we go beyond the simplistic view that “rich experiences directly lead to positive outcomes” (Feng and Johansson, 2018) and identify the critical mediating role of individual adaptability in this relationship. Specifically, functional experience diversity (FED) represents the reserve of adaptive resources accumulated by individuals, while individual adaptability denotes the dynamic capability to mobilize and deploy these resources in practical contexts. Clarifying this mediating pathway not only provides a theoretical explanation for understanding how individuals transform past career experiences into psychological advantages for coping with current challenges but also addresses why employees exhibit significantly divergent adaptive outcomes in the face of similar technological shocks.

Third, this study establishes critical contextual boundary conditions for human–robot collaboration (HRC) research. Previous studies on employee–robot collaboration have often treated the work context as homogeneous, failing to account for how task characteristics shape employees' psychological responses to technological change (Hentout et al., 2019; Charalambous et al., 2015). By introducing task type as a moderating variable and drawing on the job demands–resources (JD-R) model (Bakker et al., 2023; Cavanaugh et al., 2000), we find that the promoting effect of individual adaptability on work wellbeing is not universal but conditional. Specifically, in routine tasks, human–robot collaboration may be perceived by employees as hindrance demands. Rather than activating their adaptive capabilities, the monotonicity and low challenge of routine tasks can actually inhibit the positive effects of individual adaptability. This finding profoundly reveals the importance of the synergy among the “human–robot-task” triad and points out that the mismatch between personal resources and job demands in specific contexts is the key reason why some models of human–robot collaboration fail to enhance employees' work wellbeing.

### 6.3 Managerial implications

The findings of this study offer the following insights for managerial practice in the service industry in the context of human–robot collaboration:

First, human resource (HR) departments in the service industry should proactively build adaptive resources through recruitment and training. When recruiting for positions that will involve working with robots, managers should recognize that candidates with diverse functional backgrounds—as reflected in cross-departmental experience or multi-project involvement on their resumes—may possess greater technological resilience than those with a single professional background. For incumbent employees with low functional experience diversity, such as those who have long worked in a single position, enterprises should not only train them on how to use robots but also strategically implement job rotation and cross-departmental projects. By enriching employees' functional experience diversity, enterprises can enhance their individual adaptability, thereby fostering the growth of their work wellbeing.

Second, hotel operations departments need to achieve precision matching of human–robot collaborative tasks in job design. Managers must avoid mismatches between human resources and robotic tasks. Specifically, employees with high functional experience diversity (FED) should not be underutilized in highly repetitive human–robot collaboration positions. When an employee with high functional experience diversity (FED) and strong adaptability only engages in tasks such as monitoring robot-assisted check-in, this is not only a waste of talent but also suppresses their work wellbeing. Enterprises should redesign positions to enable them to collaborate with robots on non-routine tasks—such as complex customer complaint handling and personalized service design—thereby maximizing the synergistic value of human–robot collaboration. Meanwhile, routine tasks are more suitable for employees with low functional experience diversity (FED) to complete along with service robots.

Third, individual employees should proactively pursue diversity in their career development. This study also reminds individual employees that in the era of artificial intelligence (AI), occupational security no longer stems from specializing in a position at risk of replacement, but rather from the ability to adapt to new environments. Employees should take the initiative to apply for job rotations and participate in cross-departmental collaborations to enhance their professional resumes with functional experience diversity (FED). Doing so not only creates value for the organization but also represents a long-term investment in their own future work wellbeing and career resilience.

### 6.4 Limitations and future research

Several limitations of this study should be acknowledged, which also point to directions for future research.

First, the core constructs in this study were measured using self-report measures. Although self-reporting is an appropriate and necessary method for assessing work wellbeing (a subjective experience) and individual adaptability (an dispositional trait), relying on a single data source may introduce common method bias, potentially affecting the precision of the estimated relationships among variables. Although we implemented procedural remedies in the research design and statistical analyses—such as two-wave data collection and Harman's single-factor test—the potential influence of common method bias cannot be completely ruled out. Future research could be extended in two directions: (1) incorporating multi-source data, such as collecting supervisory or peer ratings of employees' adaptive behaviors and collaborative performance, and combining employee self-rated work wellbeing with other-rated indicators like work engagement and turnover intention, to gain a more comprehensive understanding of employees' psychological and behavioral states; and (2) adopting objective measurement methods, such as using wearable devices to capture physiological stress responses (e.g., heart rate variability, electrodermal activity), or analyzing human–robot interaction behaviors (e.g., operational efficiency, error rates, proactive learning behaviors) through system logs.

Second, this study treated service robots as a holistic concept without distinguishing between their specific types. Given that a robot's degree of anthropomorphism and level of artificial intelligence may significantly shape employees' perceptions and responses, future research should explore how these robotic characteristics moderate the proposed relationships.

Finally, employees' functional experience diversity (FED) evolves with their personal career development. Future studies could employ a longitudinal design to reveal the dynamic relationship between functional experience diversity (FED) and employees' work wellbeing.

## Data Availability

The raw data supporting the conclusions of this article will be made available by the authors, without undue reservation.
